# Independent test assessment using the extreme value distribution theory

**DOI:** 10.1186/s12919-016-0038-5

**Published:** 2016-10-18

**Authors:** Marcio Almeida, Lucy Blondell, Juan M. Peralta, Jack W. Kent, Goo Jun, Tanya M. Teslovich, Christian Fuchsberger, Andrew R. Wood, Alisa K. Manning, Timothy M. Frayling, Pablo E. Cingolani, Robert Sladek, Thomas D. Dyer, Goncalo Abecasis, Ravindranath Duggirala, John Blangero

**Affiliations:** 1 South Texas Diabetes and Obesity Institute, University of Texas at Brownsville, 2700 East Jackson Street, Brownsville, TX 78520 USA; 2 Department of Genetics, Texas Biomedical Research Institute, P.O. Box 760549, San Antonio, TX 78245-0549 USA; 3 Human Genetics Center, School of Public Health, The University of Texas Health Science Center at Houston, 7000 Fannin Street, Houston, TX 77030 USA; 4 Department of Biostatistics, Center for Statistical Genetics, University of Michigan, SPH II, 1420 Washington Heights, Ann Arbor, MI 48109-2029 USA; 5 Genetics of Complex Traits, University of Exeter Medical School, University of Exeter, Mail Room, The Old Library, Prince of Wales Road, Exeter, Devon UK; 6 Broad Institute, 415 Main Street, Cambridge, MA 02142 USA; 7 School of Computer Science & Genome Quebec Innovation Centre, McIntyre Medical Building, 903 3655 Promenade Sir William Osler McGill University, Quebec, Canada

## Abstract

The new generation of whole genome sequencing platforms offers great possibilities and challenges for dissecting the genetic basis of complex traits. With a very high number of sequence variants, a naïve multiple hypothesis threshold correction hinders the identification of reliable associations by the overreduction of statistical power. In this report, we examine 2 alternative approaches to improve the statistical power of a whole genome association study to detect reliable genetic associations. The approaches were tested using the Genetic Analysis Workshop 19 (GAW19) whole genome sequencing data. The first tested method estimates the real number of effective independent tests actually being performed in whole genome association project by the use of an extreme value distribution and a set of phenotype simulations. Given the familiar nature of the GAW19 data and the finite number of pedigree founders in the sample, the number of correlations between genotypes is greater than in a set of unrelated samples. Using our procedure, we estimate that the effective number represents only 15 % of the total number of independent tests performed. However, even using this corrected significance threshold, no genome-wide significant association could be detected for systolic and diastolic blood pressure traits.

The second approach implements a biological relevance-driven hypothesis tested by exploiting prior computational predictions on the effect of nonsynonymous genetic variants detected in a whole genome sequencing association study. This guided testing approach was able to identify 2 promising single-nucleotide polymorphisms (SNPs), 1 for each trait, targeting biologically relevant genes that could help shed light on the genesis of the human hypertension. The first gene, *PFH14*, associated with systolic blood pressure, interacts directly with genes involved in calcium-channel formation and the second gene, *MAP4*, encodes a microtubule-associated protein and had already been detected by previous genome-wide association study experiments conducted in an Asian population. Our results highlight the necessity of the development of alternative approached to improve the efficiency on the detection of reasonable candidate associations in whole genome sequencing studies.

## Background

The new generation of sequencing platforms has dramatically altered the genetics field by allowing a cost-effective approach to completely explore the information stored in a genome of interest. Large genetic and epidemiologic projects are beginning to utilize whole genome sequencing (WGS) to identify genetic variants (especially novel rare variants) that could explain the missing heritability paradox [1]. The paradox had been formulated during the genome wide association (GWA) era; those platforms are based on the CDCV (common disease common variant) premise. GWA studies have shown modest success for the identification of causal genetic variants associated with common diseases with high social impact, such as diabetes, hypertension, and cancer. The WGS approach tries to fill this large information gap by capturing all available genetic variations, including the novel, rare, and private variants that only appear in a specific family. Despite the substantial promise and potential, many statistical and analytical challenges still need to be surpassed for the efficient analysis of WGS data for the identification of causal genes.

The astronomical number of independent hypotheses tested on a WGS study, above 10 million tests, incurs substantial costs in terms of statistical power. Clearly, only a small subset of sequence variants is molecularly functional and even fewer are phenotypically functional with regard to a focal trait of interest. The assessment of the LD (linkage disequilibrium) between single-nucleotide polymorphisms (SNPs) in a WGS project is challenging because of the immense number of rare variants identified, the potential for cross-chromosome gametic disequilibrium, and, consequently, the immense number of possible pairwise correlations. A reduction in the number of relevant hypotheses tested is possible by incorporating prior knowledge of functional potential or localization of genetic variants on a target gene of interest. Many different computational approaches have been proposed for a fast and accurate prediction of the functional outcome of nonsynonymous variants. The prior selection of variants with a common functional classification of interest can directly reduce the number of tests carried out. It leads to a biologically informed hypothesis-driven exploration that should yield simpler interpretation of observed results. The addition of biologically relevant databases such as GO (Gene Ontology) or KEGG (Kyoto Encyclopedia of Genes and Genomes) greatly improve the interpretation of results gathered in a WGS association project [1].

In this report, we applied 2 distinct methods to increase the statistical power of our analysis by reducing the hypothesis state space of the association analysis performed. The first estimates the number of effective independent tests performed in WGS (or any subset of the genome) by fitting the observed extreme *p* values to a beta distribution following the method proposed by Dudbridge and Gusnanto [2]. The method is based on the extreme value distribution theory and estimates the effective number of tests parameter from a beta distribution β(1,n_e_) where *n*_*e*_ is the effective number of tests [3]. The estimate of *n*_*e*_ is the parameter *β* in the beta distribution and the best parameter is defined by a maximum likelihood approach. This method empirically controls for the redundancy that is inherent in correlated hypotheses, such as the testing of sequence variants that are in linkage/gametic disequilibrium without explicitly requiring estimation of the pairwise correlations. The second general tested approach was the reduction of the hypothesis state space using computational mapping to identify molecularly functional nonsynonymous variants. Using this subset of variants, 2 new significance thresholds were defined: the Bonferroni multiple hypothesis correction, and the second a LD corrected multiple hypothesis correction using the data generated for the first approach. Both methods were tested using the set of real systolic and diastolic phenotypes provided for Genetic Analysis Workshop 19 (GAW19) and 2 very promising associations were identified. Those nonsynonymous variants have the potential to disrupt the proteins coded by the genes *PFH14* and *MAP4*. Both genes are promising candidates and support our method to improve the statistical power of a highly dimensional WGS association project.

## Methods

### Data acquisition and quality filtering

All our analyses were performed using the GAW19 WGS family data set. A group of 6538591 SNPs with more than 5 copies of the minor allele counts (MACs) had been filtered from the initial set of 8,348,674 genetic variants. This minimum number of MACs is required to achieve approximate asymptotic test statistic expectations under the null hypothesis and was chosen based on a previously performed computer simulation. The large set of variants filtered was subdivided in groups of 500 SNPs to allow an efficient process parallelization and the files generated organized locally in our server.

### Creation of an extreme value distribution and definition of the number of effective independent tests

We simulated 1000 quantitative phenotypes under the null hypothesis of no genetic effects (h^2^ = 0). For each simulated phenotype, we performed full WGS-based association analyses for all single-nucleotide variants (SNVs) filtered in the last item. All association analyses were performed using the linear mixed model implemented in SOLAR (Sequential Oligogenic Linkage Analysis Routines), allowing for residual nonindependence between relatives [4]. The observed association results for each replicate were sorted and the most extreme association (*p* value) extracted. The distribution of 1000 extreme *p* values was fit to a theoretical beta distribution and maximum likelihood estimation was used to estimate the β parameter that is directly related to the effective number of tests. This corrected number of effective tests is used to define a new multiple hypothesis Bonferroni correction in the form of target $\alpha =0.05/ nef$ where *nef* is the number of effective tests defined by the β parameter.

### Definition of nonsynonymous variants

The genomic localization information (chromosome and position), the corresponding reference genome, and the alternative allele of each variant were jointly used as an input for the ANNOVAR (Annotate Variation) annotation tool [5]. All annotation was done using the corresponding hg19 gene annotation table and the prediction functional outcome of the nonsynonymous variants was determined using PolyPhen [6] and SIFT (sorting intolerant from tolerant) [7]. A set of 28,292 nonsynonymous SNPs was identified in the WGS data provided.

### Association analysis using the real phenotypic data

The measurements of systolic and diastolic blood pressure (SBP_1 and DBP_1) were normalized using an inverse Gaussian procedure. In all analyses, we included the covariate effects of smoking status, blood pressure medication usage, sex, age, age^2^, sex*age, sex*age^2^, and 3 principal components extracted from the genotypic dosage information (using unrelateds only) to account for latent population structure.

## Results

### Estimation of the effective number of tests

The GAW19 WGS was obtained and initially we filtered 6,538,591 SNPs with 5 or more copies of the alternative allele. A set of 1000 simulated phenotypes were generated under a null hypothesis of no genetic association with no heritability estimate. The filtered SNVs had their association with the simulated phenotypes determined using a linear mixed-model approach implemented in SOLAR. For each phenotypic simulation, the set of *p* values were sorted and the most extreme association was determined. Using the approach proposed by Dudbridge and Gusnanto in 2008 [2], we estimated the effective number of tests that maximized the fit of the set of extreme *p* value to a theoretical beta distribution. Based on this procedure, we estimated that there are only 947,109 effectively independent tests performed, corresponding to an astounding reduction of 85 % in the number of performed tests. The relatively small number of founder individuals strongly influences this reduction. This new estimate was employed to calculate a statistically appropriate multiple hypothesis significance threshold of 5.27 × e^−8^. Given the large number of complete WGS association analyses (n = 1000) performed, we also checked if the effective number of tests was consistent across the totality of the empirical null distribution. The fit appeared to be excellent suggesting that a theoretical beta distribution recapitulate the most important parameters features from the null distribution of extreme *p* values.

**Fig. 1:**
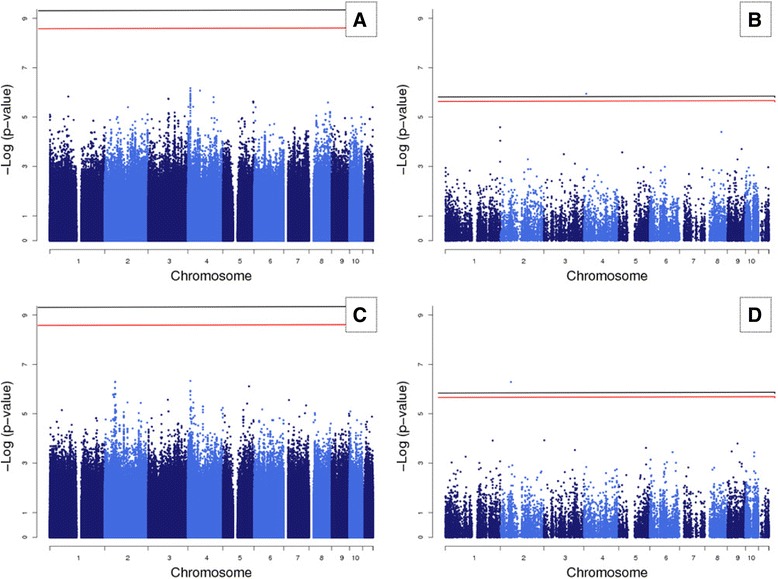
Manhattan plot representations. **a** WGS association analysis of the trait SBP; **b** nonsynonymous variants and their association with SBP; **c** WGS association analysis of the trait DBP; **d** nonsynonymous variants and their association with DBP. The black lines represent the Bonferroni multiple hypothesis threshold and the red lines the adjusted new corrected multiple hypothesis

Using the provided real SBP and DBP data, we tested the association of each of the 6.5 M SNVs using smoking status, blood pressure medication usage, sex, age, age^2^, sex*age, sex*age^2^, and 3 principal components as covariates. All associations were obtained using the linear mixed model implemented in SOLAR and interpreted using our previous simulation results (Fig. 1). The set of SNVs was tested for association using the new corrected multiple hypothesis significance threshold (red line) compared to the naïve Bonferroni correction, taking into account all SNVs in the experiment (black line). The association results for SBP are shown in Fig. 1a; the plot clearly shows that no association was identified for this trait. The top association observed for SBP was detected in the SNP rs218982 (*p* value <6.81 e^−07^) located in a nonconserved region 10 kb upstream of the gene *PHF14* in chromosome 7. The association results for DBP are shown in the Fig. 1c; and such as SBP, no clear association would be detected (Fig. 1d). The top association observed for DBP was detected in the SNP rs28429841 (*p* value <4.70 e^−07^), a rare variant with only 16 copies of the minor allele. That genetic variant is located in an intronic sequence of the gene *MAP4* in chromosome 3.

### Hypothesis reduction using prior biological information

The results in the last item explain the necessity of alternative procedures to further reduce the search space and improve the statistical power of a WGS association experiment. To accomplish such task, we reduce the search space by mapping each SNV in the latest human genome assembly (hg19) and identify a set of 28,292 nonsynonymous SNVS. This specific type of genetic variation is the cream of the crop in an association study because it directly pinpoints a candidate gene, simplifying the interpretation of the results. As expected, this reduced hypothesis test state space requires less statistical evidence for association. The Bonferroni multiple hypothesis correction in this set requires a target alpha for a genome-wide significance of only 1.76 × e^−6^, an approximately 100 times higher statistical threshold than the one required for a whole genome association. The nonsynonymous SNVs were a subset from the simulation results and the *p* values were fitted into an extreme value distribution, leading to the estimate of 13,399 independent tests. This reduced set of independent tests required a corrected new multiple hypotheses significance threshold of 3.73e^−6^. These thresholds were applied to the association results for SBP and DBP. Two SNVs, 1 for each trait, would be considered associated (see Fig. 1b and d). Interestingly, the genes carrying the most significant associated SNVs detected in the hypothesis-free WGS analysis, using all identified, SNVs were also detected in the nonsynonymous analysis.

The first SNV, rs218966, leads to a missense mutation (K115R) located in the second exon of the gene *PHF14* on chromosome 7 and shows a significant association with SBP (*p* value <1.13 × e^−6^). This gene encodes a zinc finger class transcription factor that controls the expression of the *PCBD2* gene that had been involved in the calcium channels formation [8]. Calcium channels are considered classical candidates to explain the development of pulmonary arterial hypertension and calcium-channel blockers are prescribed for treatment [9]. This variant was predicted to be benign by PolyPhen algorithm [6]. The second nonsynonymous SNV (rs9836027) identified is rare, is a missense mutation (E118K) that affects the gene *MAP4,* and is strongly associated with DBP (*p* value <5.1e^−7^). The SNV is located in an alternative use exon of the *MAP4* gene and the genetic variant is present in 2 of the 8 known gene isoforms. The *MAP4* gene encodes a microtubule-associated protein that is central to the control of cellular cycle. SNVs in this gene had already been associated with pulse pressure and mean arterial pressure in Asian individuals [10]. The association of this specific variant with DBP is novel and the functional impact of this nonsynonymous variant was predicted to be tolerable by PolyPhen, but deleterious using SIFT [7]. This discordance outlines the necessity of a further in vivo experimental validation to understand exactly the role of these genetic associations in a larger human hypertension paradigm.

## Discussion and conclusions

WGS platforms are becoming more cost effective and their application in large human pedigree studies, like San Antonio Family Studies (SAFS), allows the assessment of rare variants that are population, or even family, specific. The number of genetic variants identified in such projects is astronomical, and the required multiple hypothesis threshold correction reduces the statistical power. In this report, we used the WGS data provided by the GAW19 organization to test 2 alternative approaches to improve the statistical power of a WGS association study and favor the detection of reliable candidate genes. The first approach efficiently eliminates the redundancy of linkage/gametic disequilibrium among SNVs by the use of the extreme value theory. The estimation of LD in a dense panel of genetic marker is challenging due to a result of the required n^2^/2 computational complexity. The approximation of the extreme value distribution to a theoretical beta distribution was proposed by Šidák in 1967 and their applicability in association studies was proposed by Dudbridge and Gusnanto in 2008 [2]. The number of effective independent tests carried was reduced from 6.5 M to less than 1 M (an 85 % reduction), resulting in the statistical power improving by the same proportion. The Bonferroni correction and the newly proposed corrected multiple hypothesis correction thresholds were applied to the association results of systolic and diastolic blood pressure (see Fig. 1a and c). No clear associations would be detected for both traits. The second approach uses prior biological prediction to formulate an overarching hypothesis that is biased toward the identification of SNVs with functional potential impact. The nonsynonymous variants offer a targeted hypothesis toward the identification of candidate genes by simplifying the results annotation. We identified a set of 28,292 nonsynonymous SNVs in the GAW19 data. The dependence between nonsynonymous SNVs was eliminated by the use of the extreme value theory and a new multiple hypothesis corrected significance threshold of 3.73 × e^−6^ was defined. This procedure led to the identification of 2 missense SNVs significantly correlated with human blood pressure. The gene *MAP4* was previously associated with blood pressure alterations in Asian populations and the identified nonsynonymous SNV requires further functional validation. Our analyses highlight the necessity of the development of alternative methods favoring the detection of reasonable candidate genes in a WGS paradigm.
